# Extracellular vesicles and insulin‐mediated vascular function in metabolic syndrome

**DOI:** 10.14814/phy2.15530

**Published:** 2023-01-03

**Authors:** Tristan J. Ragland, Emily M. Heiston, Anna Ballantyne, Nathan R. Stewart, Sabrina La Salvia, Luca Musante, Melissa A. Luse, Brant E. Isakson, Uta Erdbrügger, Steven K. Malin

**Affiliations:** ^1^ Department of Kinesiology & Health Rutgers University New Brunswick New Jersey USA; ^2^ Department of Internal Medicine, Pauley Heart Center Virginia Commonwealth University Richmond Virginia USA; ^3^ Department of Kinesiology University of Virginia Charlottesville Virginia USA; ^4^ Cardiovascular Research Institute Mount Sinai New York New York USA; ^5^ School of Veterinary Medicine University of Pennsylvania Philadelphia Pennsylvania USA; ^6^ Robert M Berne Cardiovascular Research Center University of Virginia School of Medicine Charlottesville Virginia USA; ^7^ Department of Molecular Physiology and Biophysics University of Virginia School of Medicine Charlottesville Virginia USA; ^8^ Division of Nephrology, Department of Medicine University of Virginia Charlottesville Virginia USA; ^9^ Division of Endocrinology, Metabolism & Nutrition Department of Medicine New Brunswick New Jersey USA; ^10^ The New Jersey Institute for Food, Nutrition and Health Rutgers University New Brunswick New Jersey USA; ^11^ Institute of Translational Medicine and Science Rutgers University New Brunswick New Jersey USA

**Keywords:** augmentation index, inflammation, insulin resistance, obesity, pulse wave analysis, vasodilation

## Abstract

Metabolic Syndrome (MetS) raises cardiovascular disease risk. Extracellular vesicles (EVs) have emerged as important mediators of insulin sensitivity, although few studies on vascular function exist in humans. We determined the effect of insulin on EVs in relation to vascular function. Adults with MetS (*n* = 51, *n* = 9 M, 54.8 ± 1.0 years, 36.4 ± 0.7 kg/m^2^, ATPIII: 3.5 ± 0.1 a.u., VO_2_max: 22.1 ± 0.6 ml/kg/min) were enrolled in this cross‐sectional study. Peripheral insulin sensitivity (M‐value) was determined during a euglycemic clamp (40 mU/m^2^/min, 90 mg/dl), and blood was collected for EVs (CD105+, CD45+, CD41+, TX+, and CD31+; spectral flow cytometry), inflammation, insulin, and substrates. Central hemodynamics (applanation tonometry) was determined at 0 and 120 min via aortic waveforms. Pressure myography was used to assess insulin‐induced arterial vasodilation from mouse 3rd order mesenteric arteries (100–200 μm in diameter) at 0.2, 2 and 20 nM of insulin with EVs from healthy and MetS adults. Adults with MetS had low peripheral insulin sensitivity (2.6 ± 0.2 mg/kg/min) and high HOMA‐IR (4.7 ± 0.4 a.u.) plus Adipose‐IR (13.0 ± 1.3 a.u.). Insulin decreased total/particle counts (*p* < 0.001), CD45+ EVs (*p* = 0.002), AIx75 (*p* = 0.005) and Pb (*p* = 0.04), FFA (*p* < 0.001), total adiponectin (*p* = 0.006), ICAM (*p* = 0.002), and VCAM (*p* = 0.03). Higher M‐value related to lower fasted total EVs (*r* = −0.40, *p* = 0.004) while higher Adipose‐IR associated with higher fasted EVs (*r* = 0.42, *p* = 0.004) independent of VAT. Fasting CD105+ and CD45+ derived total EVs correlated with fasting AIx75 (*r* = 0.29, *p* < 0.05) and Pb (*r* = 0.30, *p* < 0.05). EVs from MetS participants blunted insulin‐induced vasodilation in mesenteric arteries compared with increases from healthy controls across insulin doses (all *p* < 0.005). These data highlight EVs as potentially novel mediators of vascular insulin sensitivity and disease risk.

## Graphical abstract

This study investigates the effects of insulin on extracellular vesicles (EVs) in relation to vascular insulin sensitivity in adults with MetS. Our data suggest that insulin infusion decreases subtypes of EVs and lowers aortic waveforms. Using pressure myography, we also show enriched EVs preps from MetS participants blunt the vasodilatory effect of insulin compared with increases from healthy controls. Thus, EVs appear to be important modifiers of vascular insulin sensitivity.

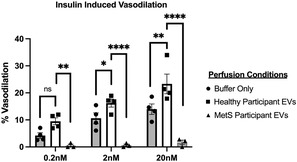

## 1 INTRODUCTION

Obesity has placed strains on our healthcare system due to, in part, increased type 2 diabetes (T2D), metabolic syndrome (MetS), and cardiovascular disease (CVD) risk (Cawley et al., 2021; Hruby & Hu, 2014; Kyrgiou et al., 2017; Xu et al., 2018). Excess adiposity, particularly in the abdominal region, contributes to this decreased quality of life through increases in multi‐organ insulin resistance and inflammation (Clerico et al., 2011; Coelho et al., 2013; Huang & Xu, 2021; Li et al., 2017; Oh et al., 2016; So et al., 2014). Extracellular vesicles (EVs) released from several cells (e.g., endothelial cells, platelets, leukocytes) (Huang & Xu, 2021; Isaac et al., 2021) modulate cellular responses to physiological stress in an autocrine, paracrine, and endocrine fashion (Akbar et al., 2019). Interestingly, fasting total circulating EVs are reported to be elevated in people with obesity compared to healthy weight individuals, suggesting a potential role in chronic disease progression (Akbar et al., 2019). The mechanism by which EVs modulate chronic disease risk is an area of intense investigation, and it has been proposed that certain subtypes of EVs downregulate insulin action in the liver, skeletal muscle, and adipocytes (Choi et al., 2015; Kranendonk, Visseren, van Herwaarden, et al., 2014; Mleczko et al., 2018).

To date, few data exist illuminating the role of EVs on vascular insulin sensitivity in humans. This is clinically relevant as insulin not only stimulates glucose uptake, but also promotes vasodilation/reduces augmentation index (AIx75) (Dotson et al., 2021), thereby contributing to lower T2D and CVD risk. In vitro data suggest that EVs may directly modify insulin action by reducing GLUT‐4 translocation as well as vasoreactivity, in part, through adipose‐derived (i.e. adipokines) inflammatory mechanisms (Pandey et al., 2019; Zhang et al., 2015). This is consistent with work our group has conducted showing that EVs are decreased in relation to circulating post‐prandial insulin levels as well as AIx75 following an oral glucose tolerance test (OGTT) in individuals with obesity and prediabetes (Eichner et al., 2019). Additionally, we recently demonstrated that insulin infusion during a euglycemic clamp decreased EVs in conjunction with metabolic insulin sensitivity before and after exercise in individuals with obesity (Heiston, Ballantyne, la Salvia, et al., 2022; Heiston, Ballantyne, Stewart, et al., 2022). However, it remains unknown if insulin modifies EVs in association with central hemodynamics, and whether body fat, adipose insulin resistance (Adipose‐IR) and/or adipokines uniquely relate to the effect of insulin on EVs. Therefore, the primary purpose of this study was to test the hypothesis that insulin‐stimulated decreases in EVs correspond with reductions in central hemodynamics. Secondarily, we examined the relationship among EVs to body fat, Adipose‐IR, adipokines, and physical activity patterns. Lastly, we compared the effects of EVs from a healthy control and MetS individuals on insulin‐stimulated vasodilation using pressure myography as an ex vivo study, as proof of concept that EVs from differing health statuses play a role in vascular function.

## 2 METHODS

### 2.1 Participants

Adults with MetS were recruited from the Charlottesville, VA area. Individuals were included in the study if they were 40–70 years, sedentary (<60 min/wk of exercise), non‐smoking without history of cardiovascular, renal, pulmonary and/or hepatic conditions, as well as, had at least 3 of 5 ATP III criteria (e.g. blood pressure, glucose, HDL, TG and/or waist circumference) (National Cholesterol Education Program (NCEP) Expert Panel on Detection, Evaluation, and Treatment of High Blood Cholesterol in Adults (Adult Treatment Panel III), 2002) and metabolic severity was calculated via a *Z*‐score as previously performed by our group (Dotson et al., 2021), as follows: *Z*‐score_male_ [(40 − HDL)/8.2] + [(TG − 150)/68.5] + [(FG − 100)/11.1] + [(WC + 102)/11.4] + [(MAP − 100)/9.0] and *Z*‐score_female_ [(50 − HDL)/8.7] + [(TG − 150)/68.5] + [(FG − 100)/11.1] + [(WC + 88)/9.6] + [(MAP − 100)/9.0]. Individuals were excluded if taking medications known to affect blood glucose (e.g. metformin, insulin, etc.). Participants underwent medical examination that included a resting and exercise electrocardiograph (ECG) as well as urine and blood chemistry analyses. All participants provided verbal and written consent to participate in the study per Rutgers and University of Virginia IRB (# Pro2020002029 and # 21244, Clinical Trials Registry # NCT04817787). Four healthy individuals (*n* = 1M/3F) between the ages of 30 and 55 years, body mass index (BMI) <25 kg/m^2^, and ATP III criteria <2 were also consented for EV collection and isolation for proof‐of‐concept portion testing EVs on ex vivo murine arterial reactivity.

### 2.2 Anthropometrics and body composition

Height and weight were collected using a stadiometer and scale to determine BMI. Waist circumference was measured two times using a measuring tape 2 cm above the navel and the averaged. A third measurement was taken if there was a difference >0.5 cm between the first two measurements. Body fat, fat‐free mass (FFM), and VAT were analyzed using dual‐energy x‐ray absorptiometry (DXA, Horizon System; Hologic).

### 2.3 Fitness and non‐exercise physical activity

Aerobic fitness (VO_2_max) was determined using indirect calorimetry (Carefusion; Vmax Encore) during an incremental treadmill test. Heart rate (HR) was measured via a 12‐lead ECG. The test was considered maximal effort if at least 3 of the following were met: HR ± 10 bpm of age‐predicted max (220‐age), rating of perceived exertion (RPE) ≥17, RER ≥1.1, and <150 ml/min change in VO_2_ with an increase in workload. Participants were also given a three‐axis accelerometer (Actigraph) and instructed to wear the device on the right hip for 7 days upon rising in the morning and remove the device before retiring to bed. A valid wear time for this measurement was considered a total of 4 days worn with an average of 10 h/day. Daily physical activities were estimated on the number of movements or counts/minute (CPM) and cut points for activity intensity (Varona et al., 2019).

### 2.4 Metabolic control

Participants were provided a diet 24 h prior to the clamp consisting of approximately 55% carbohydrate, 15% protein, and 30% fat. Caloric intake was estimated from resting metabolic rate (RMR). RMR was determined after an overnight fast in the supine position for 30 min using indirect calorimetry. Data were averaged over the last 5 min and multiplied by an activity factor of 1.2. Participants were also instructed to refrain from strenuous physical activity/exercise, medications, caffeine, and alcohol for 24 h prior to testing.

### 2.5 Hyperinsulinemic‐euglycemic clamp

Participants arrived at the Clinical Research Unit between 06:00–08:00 following an overnight fast for determination of EVs and insulin sensitivity. An intravenous line was placed in the antecubital vein for infusion of insulin and glucose. A second intravenous line was placed in the wrist/forearm veins for collection of analytes. These included: insulin, glucose, and FFAs along with total and high molecular weight (HMW)‐adiponectin and leptin as well as vascular inflammation markers ICAM (intercellular adhesion molecule 1), VCAM (vascular cell adhesion molecule 1), MMP‐1 (matrix metalloproteinase 1), MMP‐7 (matrix metalloproteinase 7). ICAM, VCAM, MMP‐1, and MMP‐7 were analyzed as they are linked to atherosclerosis, coronary artery disease, and CVD (Varona et al., 2019). This would enable understanding of EVs with vascular versus adipose‐derived inflammation. Insulin (Humulin R U‐100; Eli Lilly and Company) was diluted in saline containing the participant's blood and a primed (250 mU/m^2^/min) constant (40 mU/m^2^/min) infusion of insulin was provided for 120 min. Blood glucose was collected every 5 min and dextrose (20%) was administered at variable rates to maintain a blood glucose of 90 mg/dl. Peripheral insulin sensitivity was determined by averaging glucose metabolized between 90 and 120 min (M‐value). HOMA‐IR (Matthews et al., 1985) was calculated by multiplying fasting glucose and insulin concentrations and dividing by the constant 22.5 to estimate hepatic insulin resistance. Adipose‐IR (Malin et al., 2014) was calculated by multiplying fasting insulin with fasting FFA concentrations.

### 2.6 Central and peripheral hemodynamics

Aortic wave forms and pulse wave analysis (PWA) were conducted at minute 0 and 120 of the clamp via applanation tonometry (SphygmoCor® XCEL system; AtCor Medical) to determine vascular function responses to insulin. A blood pressure cuff was placed on the upper left arm and measurements were averaged from three trials over 10 min. Measurements were recorded while individuals rested quietly in a semi‐supine in a temperature‐controlled room. Augmentation index corrected for heart rate of 75 beats/min (AIx75), augmentation pressure (AP), brachial systolic (bSBP) and diastolic blood pressure (bDBP), central systolic (cSBP) and diastolic blood pressure (cDBP), as well as brachial and central pulse pressure (bPP and cPP) were measured. Central forward pressure (Pf), backward pressure (Pb), and reflection magnitude were also characterized through deconvolution analysis.

### 2.7 Biochemical analysis

Glucose was collected in lithium heparin capillary tubes, centrifuged at 1107 *g* for approximately 15 s and analyzed via YSI (YSI Instruments 2700). Whole blood was collected for insulin and FFAs into EDTA vacutainers containing aprotinin. These markers were collected via whole blood in serum vacutainers and allowed to coagulate for 30 min before centrifugation. Vacutainers were centrifuged at 1107 *g* for 10 min and stored at −80°C until later batch analysis. Insulin was analyzed by radioimmunoassay (Millipore), while FFAs were assessed by colorimetric assays (Wako Chemicals). Inflammation was tested using ELISA kits (R&D Systems, Inc.).

### 2.8 EV collection and preparation for characterization

Blood was collected at 0 and 120 min of the clamp in citrate vacutainers and underwent differential centrifugation at 5000 *g* for 15 min (Sorvall RC 5B Plus Centrifuge: Rotor SS‐34 Fixed Angle Rotor). The platelet poor plasma was then transferred to 1.5 ml microcentrifuge tubes (Axygen) and frozen at −80°C. Prior to analysis, blood was thawed and centrifuged at 17,000 *g* for 20 min (called P17 pellet) (centrifuge: 524/4324 R‐Rotor FA‐45‐24‐11; Eppendorf) and fluorescently labeled with antibodies for EV phenotyping by spectral flow cytometry. 6 μl of Ab mix and 4 μl of HEPES buffer was added to the pEV solution for a total final incubation volume of 30 μl. An antibody titer was performed for each antibody used and the following amount was optimized in the cocktail: 0.25 μl (CD9/CD81/CD63 tetraspanin cocktail; BioLegend); 7 μl (CD45); 5 μl (CD105/CD31/CD41; BioLegend). The EV sample (30 μl) was then stained with 20 μl of buffer plus reagents for 1 h in the dark at room temperature. After staining, each sample was washed in 0.1 μM HEPES PBS at 21,000 *g* for 1 h and resuspended in 50 μl. Before flow acquisition, each sample was diluted in different volumes (D:500; D:1000; D:2000) according to MISEV (Minimal information for studies of EVs) guidelines as done recently per our group (Eichner et al., 2019; Heiston, Ballantyne, la Salvia, et al., 2022; la Salvia et al., 2020; Théry et al., 2018). We also followed the MIFlowCyt‐EV guidelines as outlined by our group before (Welsh, van der Pol, et al., 2020) (Table S1). We have also provided our gating strategy and dilution experiments (Figures S1 and S2).

### 2.9 EV classification and phenotyping

Basic EV characterization was performed using cryo‐electron microscopy (cryo‐EM) for EV morphology analysis, NTA for EV count and sizing, and Western blotting (WB) for detection of EV and non‐EV related proteins as described previously (Heiston, Ballantyne, la Salvia, et al., 2022). EVs were labeled and classified by flow cytometry as: Total EV/particle counts (TPC), endothelial‐ (CD105+), leukocyte‐ (CD45+), platelet‐ (CD41+), and tetraspanin‐derived EVs (TX+), as well as platelet endothelial cell adhesion molecule positive EVs (CD31+). EVs were identified, labeled, and counted using a spectral flow cytometer (Cytek® Aurora 5) with a small particle enhancement modification (Heiston, Ballantyne, la Salvia, et al., 2022). EV concentrations were calculated using a volumetric method provided by the full spectrum flow cytometer (FSFC) utilizing herein. Of note, information about total EV/particle counts (TPC) <1000 nm in size, which can contain other EV particles was also collected. Scatter intensity data were calibrated in standard units of scattering cross‐section (nm2) and diameter (nm), which allowed for specific phenotype analysis of two ranges of EV size: (medium sized 200–625 nm: large size EVs: 625–1000 nm) (Heiston, Ballantyne, la Salvia, et al., 2022). Size and Molecules of Soluble Fluorescence (MESF beads, Bangs Laboratories, Inc) data were generated using FCM_PASS_ Software (Welsh, Horak, et al., 2020). This software performed a particle diameter approximation using Mie Theory and NIST traceable polystyrene particles of known size and refraction index (RI). Following the input of scatter intensity values of the calibration particles, size distribution curves were generated. Diameters were then extrapolated using raw SSC data and particle diameter distributions were produced. Likewise, calibrated units of MESF were extrapolated from the arbitrary units (a.u.) of fluorescence intensity reported by the FSFC using intensity of calibrated fluorescent particles.

### 2.10 Human EVs and murine arterial reactivity

Our previous publication provides detailed EV testing of rodents and humans with pressure myography (Good et al., 2020). Of note, we could reproduce the work from rodent‐derived EVs in rodent vessels using human‐derived EVs. For that reason, we were confident to test human‐derived EVs in mice vessels in this study. Freshly isolated 3rd order mouse (Male C57Bl/6; obtained from Jackson Laboratory ages 10–15 weeks) mesenteric arteries (100–200 μm) were mounted in a pressure arteriography (Danish MyoTechnology [DMT]) and equilibrated for 30 min at 80 mmHg and 37°C. As proof‐of‐concept, EVs isolated from 1 ml of blood from adults with MetS (*n* = 3) and healthy control participants (*n* = 4). EVs from the MetS and healthy participants were then added to one mouse mesenteric vessel. This amount of EV is sufficient to restrain vasodilation in WKY by use of EVs from normotensive WKY as described before (Good et al., 2020). EVs from the P17 pellet (see preparation for EV phenotyping with flow cytometry) were resuspended in 100 μl PBS buffer. We are aware that differential centrifugation (DCF) is only providing an enriched EV prep. While we cannot rule out that other non‐EV proteins are co‐sedimented with this EV enrichment method, we decided to use the same prep (DCF) which we used for flow cytometry. In order to perform cleaning steps with size exclusion chromatography and/or density gradient we would have needed at least 10–15 times more samples. Future studies are needed to understand the effect of “pure EVs” and their cargo. The 3rd order mesenteric mice arteries were cannulated on a pressure myograph and pressurized to 80 mmHg. EVs (~6 × 10^7^ EV/ml, average of EVs per 1 ml of healthy samples counted by NTA) were perfused through the vessel lumen and circulated in bath solutions and equilibrated for 10 min. Inner diameter was measured with digital calipers in response to cumulative concentrations of insulin (0.2, 2, and 20 nM) to determine insulin‐stimulated effects on arterial reactivity. Confirmation of vessel health was assessed using 1uM NS309 (Endothelial check) and 30 mM KCl (smooth muscle check). Vessels that did not respond to these drug treatments were excluded from data analysis.

### 2.11 Statistical analysis

Data were analyzed with SPSS (IBM 26th Edition). Normality was determined and non‐normality data were log transformed for analysis. Two‐tailed paired *t*‐tests were used to assess the effect of insulin infusion on outcomes between 0 and 120 min of the clamp as well as differences between EV medium‐ and high‐scatter size. For pressure myography, two‐way ANOVA with a Holm‐Sidak's multiple comparisons test were used to understand insulin‐stimulated arterial reactivity. Pearson correlations assessed significant relationships and VAT served as a co‐variate in bivariate regression when assessing relationships between insulin sensitivity, metabolic syndrome severity and EVs. Cohen's *d* effect size was also calculated to assess physiological relevance with small = 0.2, medium = 0.5, and large = 0.8, respectively. Data are reported as mean ± SEM. Statistical significance was accepted as *p* ≤ 0.05.

## 3 RESULTS

### 3.1 Participant characteristics

Fifty‐one adults (54.8 ± 1.0 years) with MetS (ATP III: 3.47 ± 0.1; metabolic severity *Z*‐score 2.5 ± 0.3) completed the study, with 82% being female (*n* = 42) and 92% identified as White (*n* = 47; Table 1). Due to technical issues, only 28 individuals had valid accelerometer data available for analysis of non‐exercise physical activity. In this subgroup analysis, participants spent 77.7 ± 1.1% of their time in sedentary activities, with 18.5 ± 0.9% in light physical activity and 3.7 ± 1.4% in moderate‐to‐vigorous physical activities. The amount of time spent in vigorous activities was negligible. Together, these physical activity findings are consistent with low aerobic fitness (VO_2_max = 22.1 ± 0.6 ml/kg/min).

**TABLE 1:** Participant demographics

*N* (sex)	51 (42F/9M)
Age (year)	54.8 ± 1.0
Ethnicity & race	
White, non‐Hispanic	47 (92%)
Black, non‐Hispanic	3 (6%)
Hispanic, regardless of race	1 (2%)
Fitness and physical activity	
VO_2_max (L/min)	2.3 ± 0.1
VO_2_max (ml/kg/min)	22.1 ± 0.6
Sedentary time (%)^b^	77.7 ± 1.1
Light physical activity time (%)^b^	18.5 ± 0.9
Mod‐to‐Vigo. PHYSICAL ACTIVITY TIME (%)^b^	3.7 ± 1.4
Body composition	
Weight (kg)	104.8 ± 2.4
BMI (kg/m^2^)	36.4 ± 0.7
Total body fat (%)^a^	44.1 ± 0.8
Total fat free mass (%)^a^	55.9 ± 0.8
Visceral fat volume (cm^3^)^a^	1032.8 ± 44.0
Metabolic syndrome criteria	
Metabolic *Z*‐score (a.u.)	2.4 ± 0.3
ATP III score (a.u.)	3.47 ± 0.1
Glucose (mg/dl)	100.2 ± 2.2
HDL (mg/dl)	46.5 ± 1.3
Triglycerides (mg/dl)	150 ± 9.6
Systolic BP (mmHg)	132.7 ± 1.5
Diastolic BP (mmHg)	77.7 ± 1.3
WC (cm)	114.6 ± 1.5

*Note*: Data are mean ± SEM.

Abbreviations: BMI, body mass index; BP, blood pressure; HDL, high‐density lipoprotein; WC, waist circumference.

^a^ *n* = 41.

^b^ *n* = 28.

### 3.2 Insulin sensitivity and inflammation

Participants demonstrated low metabolic insulin sensitivity, as reflected by reduced peripheral insulin sensitivity (M‐value = 2.6 ± 0.2 mg/kg/min), elevated Adipose‐IR (13.0 ± 1.3 a.u.) and hepatic insulin resistance (4.7 ± 0.4 a.u.). Yet, circulating lactate increased (*p* < 0.001, *d* = 0.55) while FFA decreased (*p* < 0.001, *d* = 3.3) from 0 to 120 min of the clamp (Table 2). In addition, total adiponectin (*p* = 0.006, *d* = 0.43), but not HMW adiponectin (*p* = 0.84), decreased during insulin infusion. Insulin infusion also decreased both ICAM (*p* = 0.002, *d* = 0.5) and VCAM (*p* = 0.03, *d* = 0.3), despite no effect on MMP‐1 (*p* = 0.10) or MMP‐7 (*p* = 0.07; Table 2).

**TABLE 2:** Hemodynamics and blood chemistries during the euglycemic‐hyperinsulinemic clamp

	Fasted	Insulin‐stimulated	*p‐*value	Cohen's *d*
	0 min	∆ (120 − 0 min)		
Hemodynamics				
bSP (mmHg)	135.4 ± 2.4	−0.5 ± 1.8	0.779	0.04
bDP (mmHg)	81.8 ± 1.4	−1.0 ± 1.0	0.321	0.15
bMAP (mmHg)	98.7 ± 1.7	−0.9 ± 1.2	0.436	0.12
bPP (mmHg)	53.5 ± 1.6	−0.4 ± 1.3	0.729	0.05
HR (bpm)	64.3 ± 1.3	2.7 ± 0.9	0.006	0.43
cSP (mmHg)^a^	125.7 ± 2.0	−2.0 ± 1.5	0.256	0.17
cDP (mmHg)^a^	82.0 ± 1.7	−0.5 ± 1.4	0.531	0.09
cMAP (mmHg)	95.6 ± 2.0	−0.9 ± 2.6	0.880	0.02
cPP (mmHg)	42.1 ± 1.3	−1.0 ± 1.0	0.332	0.15
AP (mmHg)^a^	14.1 ± 0.9	−2.0 ± 0.8	0.001	0.50
AIx75 (%)	27.4 ± 1.4	−3.6 ± 1.2	0.005	0.44
Pb (mmHg)^a^	19.0 ± 0.6	−1.0 ± 0.5	0.044	0.31
Pf (mmHg)	28.2 ± 1.0	−0.2 ± 0.7	0.829	0.03
RM (%)	66.8 ± 1.9	−2.3 ± 2.6	0.379	0.13
Blood chemistries				
Insulin sensitivity (mg/kg/min)	2.6 ± 0.2	—	—	—
HOMA‐IR^a^	4.7 ± 0.4	—	—	—
Adipose‐IR	13.0 ± 1.3	—	—	—
Glucose (mg/dl)^b^	103.2 ± 1.6	−15.4 ± 1.8	<0.001	0.88
Lactate (mmol/L)	0.8 ± 0.03	0.1 ± 0.0	<0.001	0.55
Insulin (uU/L)	18.2 ± 1.6	64.8 ± 3.4	<0.001	2.38
ICAM (ng/ml)	216.7 ± 7.4	−10.0 ± 3.0	0.002	0.46
VCAM (ng/ml)	555.8 ± 115.1	−17.5 ± 7.9	0.031	0.31
MMP‐1 (ng/ml)	4.6 ± 0.5	0.3 ± 0.2	0.101	0.18
MMP‐7 (ng/ml)	2.8 ± 0.2	−0.2 ± 0.2	0.071	0.29
FFA (mEq/L)	0.7 ± 0.02	−0.5 ± 0.0	<0.001	3.27
Total adiponectin (ng/ml)	9483.7 ± 609.7	−463.0 ± 159.1	0.006	0.43
HMW adiponectin (ng/ml)	3810.1 ± 315.9	−31.1 ± 151.3	0.838	0.18
HMW:Total adiponectin	0.4 ± 0.0	0.01 ± 0.0	0.585	0.08
Leptin (ng/ml)	50.6 ± 4.2	1.1 ± 1.0	0.276	0.18
Total Adipo:Lep	229.1 ± 22.6	−508.5 ± 472.8	0.084	0.31
HMW:Lep	98.3 ± 15.6	−418.9 ± 258.3	0.975	0.01

*Note*: Data are mean ± SEM.

Abbreviations: AIx@75, augmentation index; bDP, brachial diastolic pressure; bSP, brachial systolic pressure; FFA, free fatty acid; HR, heart rate; ICAM, intercellular adhesion molecule 1; MAP, mean arterial pressure; Pb, backward wave pressure; Pf, forward wave pressure; PP, pulse pressure; RM, reflection magnitude; VCAM, vascular cell adhesion molecule 1.

^a^ Log transformed for statistical analysis.

^b^ Insulin‐stimulated was calculated with the average of the 90‐, 105‐ and 120‐min time points. Raw data are shown for ease of interpretation.

### 3.3 Central hemodynamics

Insulin infusion did not change bSBP, bDBP, PP, MAP, Pf, or RM. However, insulin reduced AIx75 (*p* = 0.005, *d* = 0.44), AP (*p* = 0.001, *d* = *0*.50), and Pb (*p* = 0.04, *d* = 0.31) as well as increased HR from 0 to 120 min of the clamp (*p* = 0.006, *d* = 0.43; Table 2).

### 3.4 Extracellular vesicles

Insulin infusion decreased TPC EV count from minutes 0 and 120 during insulin infusion (*p* < 0.001, *d* = 1.03; Table 3). This effect was attributed to a decrease in both medium (*p* < 0.001, *d* = 1.02) and large (*p* < 0.001, *d* = 1.03) EV sizes. Insulin specifically reduced CD+45 (*p* = 0.002, *d* = 0.45) during the clamp. However, insulin infusion had no effect on total, medium, or large concentrations of CD105+ (*p* = 0.26), TX+ (*p* = 0.14), CD31+ (*p* = 0.17), or CD41+ (*p* = 0.32).

**TABLE 3:** Extracellular vesicles (EVs) during the euglycemic‐hyperinsulinemic clamp

	Fasted	Insulin‐stimulated	*p‐*value	Cohen's *d*
	0 min	(∆ 120 − 0 min)		
TPC^a^	5.3 × 10^9^ ± 1.8 × 10^8^	−1.6 × 10^9^ ± 8.4 × 10^8^	<0.001	1.03
Medium^a^	3.9 × 10^9^ ± 8.3 × 10^8^	−1.1 × 10^9^ ± 5.6 × 10^8^	<0.001	1.02
Large^a^	1.4 × 10^9^ ± 4.0 × 10^8^	−4.6 × 10^8^ ± 2.6 × 10^8^	<0.001	1.03
CD105+ Total	1.9 × 10^7^ ± 1.5 × 10^7^	1.4 × 10^7^ ± 1.5 × 10^7^	0.259	0.16
CD105+ Medium	1.5 × 10^7^ ± 1.2 × 10^7^	−1.1 × 10^7^ ± 1.2 × 10^7^	0.353	0.13
CD105+ Large	4.6 × 10^6^ ± 2.9 × 10^6^	−2.9 × 10^6^ ± 3.0 × 10^6^	0.120	0.22
CD45+ Total^a^	1.1 × 10^7^ ± 2.3 × 10^6^	−4.0 × 10^6^ ± 2.3 × 10^6^	0.002	0.45
CD45+ Medium Size^a^	7.2 × 10^6^ ± 2.3 × 10^6^	−2.8 × 10^6^ ± 1.7 × 10^6^	0.002	0.47
CD45+ Large Size^a^	4.0 × 10^6^ ± 7.6 × 10^5^	−1.2 × 10^6^ ± 8.3 × 10^5^	0.021	0.34
CD41+ Total^a^	5.2 × 10^7^ ± 1.9 × 10^6^	1.6 × 10^7^ ± 3.4 × 10^7^	0.323	0.14
CD41+ Medium^a^	3.7 × 10^7^ ± 1.3 × 10^7^	1.7 × 10^7^ ± 2.7 × 10^7^	0.290	0.15
CD41+ Large^a^	1.5 × 10^7^ ± 5.7 × 10^6^	−8.4 × 10^5^ ± 7.0 × 10^6^	0.356	0.13
TX+ Total^a^	6.1 × 10^7^ ± 1.8 × 10^7^	7.4 × 10^6^ ± 2.7 × 10^7^	0.138	0.21
TX+ Medium^a^	4.3 × 10^7^ ± 1.2 × 10^7^	9.1 × 10^6^ ± 2.1 × 10^7^	0.132	0.22
TX+ Large^a^	1.8 × 10^7^ ± 6.1 × 10^6^	−1.7 × 10^6^ ± 7.1 × 10^6^	0.146	0.21
CD31+ Total^a^	2.8 × 10^7^ ± 8.7 × 10^6^	2.5 × 10^6^ ± 1.3 × 10^7^	0.171	0.19
CD31+ Medium^a^	1.6 × 10^7^ ± 4.6 × 10^6^	3.2 × 10^6^ ± 8.9 × 10^6^	0.127	0.22
CD31+ Large^a^	1.2 × 10^7^ ± 4.0 × 10^6^	−6.4 × 10^5^ ± 5.1 × 10^6^	0.217	0.18

*Note*: Data are mean ± SEM.

^a^ Log transformed for statistical analysis. *p*‐value and Cohen's *d* statistical analysis between 0 and 120 min. Raw data and Delta (120 − 0 min) are shown for ease of interpretation. Total EV/particle count (TPC), endothelial‐ (CD105+), leukocyte‐ (CD45+), platelet‐ (CD41+), and tetraspanin‐derived EVs (TX+), platelet endothelial cell adhesion molecule (CD31+), leukocyte‐derived extracellular vesicles (CD45+).

### 3.5 Arterial reactivity

**FIGURE 1:**
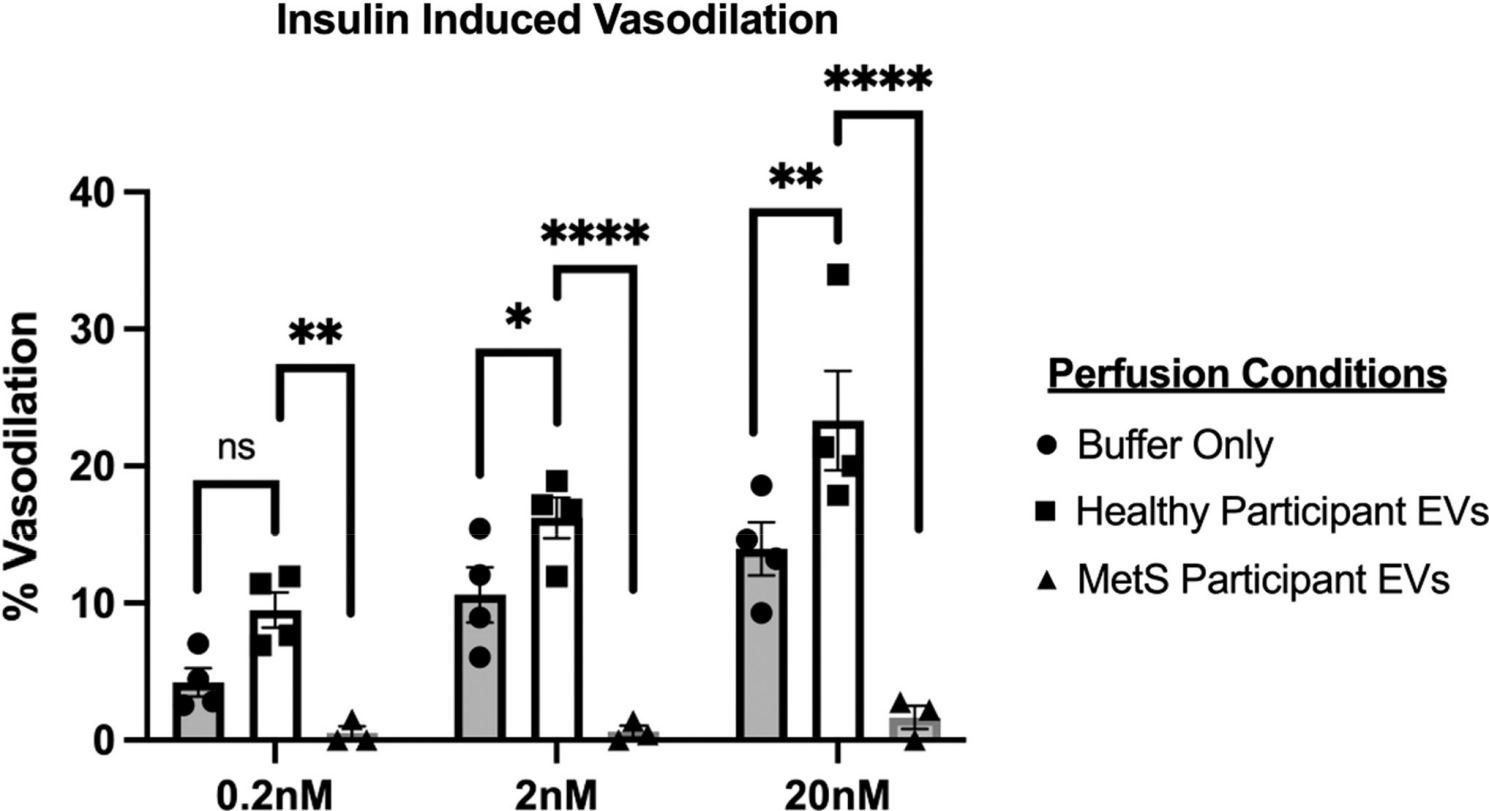
Extracellular vesicles (EVs) from healthy and MetS participants effect insulin‐stimulated vasodilation. Percent vasodilation calculated as a percentage of maximum diameter (diameter of artery in calcium free buffer) for each dose on insulin. Buffer only perfusion (circles) were performed to establish baseline levels of insulin‐induced vasodilation on vessels that have been perfused through the lumen. EVs from healthy (black square) and MetS (triangle) participants were perfused through the vessel lumen and subjected to a series of insulin doses (denoted on the *x*‐axis). Statistics include a two‐way ANOVA with a holm‐Sidak's multiple comparisons test, *p < 0.05, **p < 0.01, ****p < 0.0001.

Using 3rd order mesenteric arteries from mice, insulin alone promoted arterial vasodilation and is considered a baseline measurement of insulin induced vasodilation (Figure 1). When healthy human EVs were perfused through mouse arteries, an enhanced insulin induced vasodilation was seen. This vasodilatory effect was blunted when EVs from humans with MetS were perfused through mouse arteries across 0.2, 2, and 20 nM doses of insulin (all *p* < 0.005, Figure 1).

### 3.6 Correlations

**FIGURE 2:**
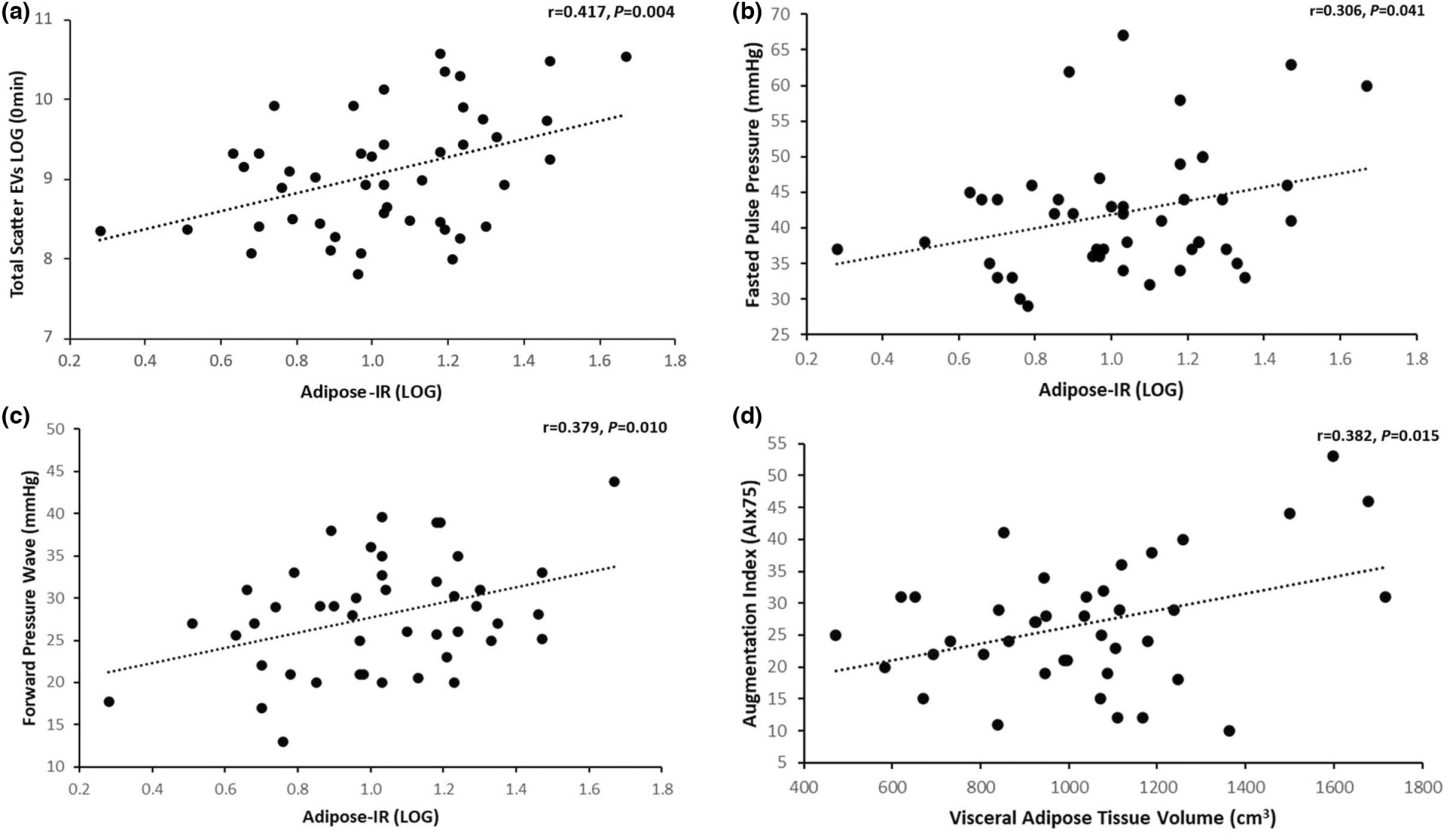
Correlations of Adipokines and visceral adipose tissue with total EVs. ^denotes log transformation to correct normality. (a) Correlation between fasted total scatter extracellular vesicles^ (TPS) to adipose insulin resistance^ (adipose‐IR), (b) fasted pulse pressure (PP mmHg) to adipose‐IR^, (c) fasted forward pressure wave (pf mmHg) to adipose‐IR^, (d) fasted augmentation index (AIx75) to visceral adipose tissue volume (VAT cm^3^).

Clamp‐derived peripheral insulin sensitivity was associated with lower fasting TPC EVs (*r* = −0.40, *p* = 0.004; Table 4), as well as lower medium (*r* = −0.4, *p* = 0.005) and large sized EVs respectively (*r* = −0.4, *p* < 0.001). Importantly, the relationship between peripheral insulin sensitivity and total (*r* = −0.35, *p* = 0.037), medium (*r* = −0.33, *p* = 0.038) and large (*r* = −0.42, *p* = 0.008) EVs were maintained when co‐varying for VAT. Peripheral insulin sensitivity was also associated with insulin mediated changes in TX+ (*r* = 0.28, *p* = 0.048) and CD41+ (*r* = 0.28, *p* = 0.049). Elevated metabolic *Z*‐score was associated with elevated fasting TPC (*r* = 0.29, *p* = 0.04), medium EV (*r* = 0.29, *p* = 0.04), and large EV (*r* = 0.34, *p* = 0.02), respectively, but this relationship appears to be attenuated by VAT for TPC (*r* = 0.24, *p* = 0.14), medium (*r* = 0.23, *p* = 0.15), and large EVs (*r* = 0.29, *p* = 0.07). Elevated HOMA‐IR related to higher fasted EVs concentrations (*r* = 0.43, *p* = 0.002) but this relationship was reduced when covarying for VAT (*r* = 0.30, *p* = 0.076). Elevated Adipose‐IR (Figure 2) related to higher fasted TPC EVs (*r* = 0.417, *p* = 0.004) even when co‐varying for VAT (*r* = 0.371, *p* = 0.028). Higher Adipose‐IR also related to elevated fasted PP (*r* = 0.306, *p* = 0.041) and Pf (*r* = 0.379, *p* = 0.010). Increased VAT was associated with high Adipose‐IR (*r* = 0.336, *p* = 0.045), fasting AIx75 (*r* = 0.382, *p* = 0.015; Figure 2) and CD105+ (*r* = 0.34, *p* = 0.027). Elevated fasted HR corresponded with elevations in TX+ (*r* = 0.35, *p* = 0.013), CD31+ (*r* = 0.34, *p* = 0.017), and CD41+ (*r* = 0.35, *p* = 0.015). Likewise, elevated fasted AIx75 related to increased CD105+ (*r* = 0.29, *p* = 0.039), and elevated Pb related to increased TX+ (*r* = 0.30, *p* = 0.035) and CD41+ (*r* = 0.30, *p* = 0.035; Table 4). Insulin‐mediated reductions in ICAM were associated with preservation and/or slight increases during insulin‐infusion with TX+ (*r* = −0.31, *p* = 0.03), CD31+ (*r* = −0.31, *p* = 0.03), and CD41+ (*r* = −0.32, *p* = 0.02; Table 4). Higher VAT also correlated with a reduction in insulin‐stimulated CD105+ (*r* = −0.359, *p* = 0.021), and CD45+ (*r* = −0.412, *p* = 0.007; Table 5). These data demonstrate the potential role of insulin‐mediating EVs as part of the crosstalk between vascular function and adiposity.

**TABLE 4:** Correlations of insulin sensitivity, vascular inflammation, and hemodynamics to Total EVs

Fasting	M‐value	AdiposeIR	HOMA IR	ICAM	VCAM	HR	AIx75	Pb
TPC	**−0.40**	**0.42**	**0.43**	0.14	−0.01	−0.03	−0.11	0.22
CD105+	−0.01	0.11	0.02	−0.07	−0.03	0.07	**0.29**	0.09
CD45+	−0.12	0.14	0.05	−0.06	0.13	0.25	0.20	0.12
TX+	−0.08	0.17	0.19	0.02	0.09	**0.35**	0.12	**0.30**
CD31+	−0.03	0.16	0.16	0.00	0.11	**0.34**	0.12	0.26
CD41+	−0.03	0.14	0.15	0.02	0.22	**0.35**	0.12	**0.30**

Insulin‐stimulated		—	—	∆ICAM	∆VCAM	∆HR	∆AIx75	∆Pb
∆TPS	0.12	—	—	−0.25	−0.18	−0.05	−0.14	−0.05
∆CD105+	0.13	—	—	0.05	−0.02	−0.03	−0.09	−0.06
∆CD45+	0.03	—	—	0.01	−0.03	−0.07	−0.16	−0.10
∆TX+	**0.28**	—	—	**−0.31**	−0.03	−0.02	−0.14	−0.02
∆CD31+	0.26	—	—	**−0.31**	−0.03	−0.02	−0.16	−0.03
∆CD41+	**0.28**	—	—	**−0.32**	−0.02	−0.01	−0.15	−0.01

*Note*: Bolded values indicate significant associations (*p* < 0.05). Total EV/particle count (TPC), endothelial‐ (CD105+), leukocyte‐ (CD45+), platelet‐ (CD41+), and tetraspanin‐derived EVs (TX+), as well as platelet endothelial cell adhesion molecule (CD31+). Intercellular adhesion molecule 1 (ICAM), vascular cell adhesion molecule 1 (VCAM), leukocyte‐derived extracellular vesicles (CD45+).

Abbreviations: AIx@75, augmentation index; bDP, brachial diastolic pressure; bSP, brachial systolic pressure; HR, heart rate; MAP, mean arterial pressure; Pb, backward wave pressure; Pf, forward wave pressure; PP, pulse pressure; RM, reflection magnitude.

**TABLE 5:** Correlations of Adipokines and visceral adipose tissue with total EVs.

Fasting	Total adiponectin	HMW adiponectin	Leptin	Total Adipo:Lep	HMW:Lep	VAT Vol (cm^3^)
TPC	−0.18	−0.26	−0.28	0.11	−0.05	0.14
CD105+	0.20	0.16	0.14	0.18	0.05	**0.34**
CD45+	0.08	0.06	0.05	0.27	0.05	0.30
TX+	−0.6	−0.21	−0.01	−0.13	−0.18	0.28
CD31+	−0.06	−0.13	−0.09	0.10	<0.01	0.30
CD41+	−0.09	−0.14	−0.12	0.09	<−0.01	0.27

Insulin‐stimulated	∆Total adiponectin	∆HMW adiponectin	∆Leptin	∆Total Adipo:Lep	∆HMW:Lep	VAT Vol (cm^3^)
∆TPC	−0.09	0.05	0.08	−0.10	−0.18	−0.17
∆CD105+	−0.12	**−0.40**	0.04	−0.14	−0.23	**−0.36**
∆CD45+	−0.10	**−0.35**	−0.02	−0.11	−0.20	**−0.41**
∆TX+	−0.08	−0.08	−0.02	0.10	−0.02	−0.24
∆CD31+	−0.10	−0.11	−0.01	0.02	−0.11	−0.24
∆CD41+	−0.09	−0.07	0.02	0.10	0.25	−0.20

*Note*: Bolded values indicate significant associations (*p* < 0.05). Total EV/particle count (TPC), endothelial‐ (CD105+), leukocyte‐ (CD45+), platelet‐ (CD41+), and tetraspanin‐derived EVs (TX+), as well as platelet endothelial cell adhesion molecule (CD31+).

Abbreviations: HMW, high molecular weight adiponectin; HWM:Lep, Adipo:Lep, HMW‐adiponectin to leptin ratio.

## 4 DISCUSSION

The primary finding from this work is that insulin significantly lowered total and leukocyte‐derived EVs in conjunction with decreased AP, AIx75, and Pb in adults with MetS. We also determined EVs from adults with MetS blunted the dilatory effect insulin has on murine 3rd order mesenteric arteries compared to the enhanced effect of EVs from healthy adults. These data demonstrate the potential for EVs to interact with insulin and modulate vascular responses. Although we observed no direct correlation between insulin‐stimulated decreases in EVs with reductions in aortic waveforms in these individuals with MetS, it remains possible that EVs modulate endothelial function or arterial stiffness through alternative assessments (e.g. FMD, PWV, etc.). However, the ex vivo approach, highlights that enriched EV preps likely affect insulin‐induced vascular function. Of note, the enriched EV preparations used for the vascular testing were obtained from humans and were therefore “*generated in vivo*”. This supports the translational aspect of this work. Other ex vivo vascular function tests have primarily used cell culture‐derived EVs (from endothelial‐ and platelet‐derived cells) (Martin et al., 2004; Otani et al., 2018; Pfister, 2004). Consistent with this hypothesis that these enriched EV preps affect insulin action, elevated fasting AIx75, Pb and HR were each significantly associated with elevated fasting CD105+, CD31+ and CD41+ EVs. These later findings are in line with our prior work in people who had obesity, with or without prediabetes (Eichner et al., 2019), whereby fasting AIx75 correlated with reductions in EVs during an OGTT. In turn, it is not surprising then that peripheral insulin sensitivity and Adipose‐IR were each significantly correlated with fasting TPC EVs independent of VAT. Although the relationship of HOMA‐IR to EVs appears to be influenced by VAT, these general findings are consistent with literature using fasting indices of insulin sensitivity that EVs are related to mechanisms that regulate insulin action (Choi et al., 2015; Eichner et al., 2019; Heiston, Ballantyne, la Salvia, et al., 2022; Kranendonk et al., 2014; Mleczko et al., 2018; Zhang et al., 2015). Indeed, the relationship with metabolic syndrome severity and EVs highlight those with healthier states may respond to insulin by reducing EVs to a greater extent. Nonetheless, our work extends upon these observations in humans by showing that clamp‐derived insulin sensitivity is also related, and EVs from humans modify insulin‐induced vasoreactivity ex vivo. Taken together, this work suggests that EVs play a role in multi‐organ insulin resistance and warrant additional work to elucidate mechanisms in which precision medical treatment may target EVs for treatment of CVD.

There are several reasons why EVs may interact with insulin action. Insulin is an anti‐inflammatory hormone, such that we (Dotson et al., 2021) and others (Dandona et al., 2007; Park et al., 2018) have shown decreases inflammation including C‐RP and MMP‐7 (Dotson et al., 2021). This is relevant as adipokines (e.g. adiponectin) and vascular markers (e.g. ICAM/VCAM) are implicated in the regulation of EV release and/or uptake (Connolly et al., 2021; Prattichizzo et al., 2021). Given that excess body fat relates to elevations in inflammation, it would seem reasonable to anticipate insulin‐mediated reductions in EV levels correspond with adiposity driven inflammation. Indeed, insulin has an anti‐inflammatory effect in some, but not all studies pertaining to people with insulin resistance (Dotson et al., 2021). Herein, adipose tissue was assessed by DXA, and we report that higher VAT was linked to higher fasting CD105+. Likewise, elevated levels of VAT corresponded with reductions in endothelial and leukocyte EVs (i.e. CD105+ and CD45+). This suggests that while increased VAT relates to fasting EVs, individuals may maintain levels of insulin responsiveness. Thus, VAT may play distinct roles in contributing to CVD risk in fasting versus insulin‐stimulated states via EVs. Another possibility is that adipokines per se, compared with fat mass, play a more direct role in modulating EV levels. In recent years, assessment of adiponectin and leptin have been used as indices for adiposapathy, or “sick fat” (Bays, 2014; Bini et al., 2021; Heiston et al., 2020). In the present work, insulin was shown to reduce total adiponectin but have no effect on HMW‐adiponectin or leptin. This is mechanically important as total adiponectin is a multi‐tier protein with low‐, medium‐, and high‐weight isoforms that independently modifies cardiometabolic health (Horakova et al., 2018). In fact, HMW‐adiponectin is considered the bioactive form that modulates insulin sensitivity and fatty‐acid oxidation (Horakova et al., 2018). Interestingly, the decrease in total adiponectin with no change in HMW adiponectin suggests that low‐to mid‐weight forms were reduced, thereby conferring potential glucose utilization benefits under insulin infusion. To corroborate this notion, preservation of HMW‐adiponectin during the clamp was inversely related to CD105+ and CD45+. Moreover, the reduction in HMW to leptin ratio (i.e. adiposapathy) was related to declines in CD105+, CD45+, CD31+ and CD41+. Collectively, these data showcase adiponectin as an important modifier of EVs under fasting and insulin‐stimulated states and future work may consider these implications toward CVD progression/treatment (Kranendonk, Visseren, van Balkom, et al., 2014).

Vascular inflammation is considered an important modulator of endothelial function and central hemodynamics (Dotson et al., 2021). In the present study, we observed a significant decrease in ICAM and VCAM in response to insulin, while MMP‐7 and MMP‐1 trended. This could be meaningful as the reduction in ICAM during the clamp related to the rise in TX+, CD31+ and CD41+ EVs. Generally, higher ICAM is associated with declines in endothelial function (Becker et al., 2000; Lawson & Wolf, 2009) and atherosclerosis (Varona et al., 2019). As such, declines in ICAM would be expected from insulin. Both CD31+ and CD41+ are platelet‐derived EVs. ICAM influences vascular integrity via endothelial cell‐to‐cell junctions (Kong et al., 2021) and leads to platelet activation. Therefore, we speculate that declines in ICAM during insulin stimulation may reduce activation of platelet‐derived EVs in comparison to the fasting state, such that preservation of said EVs are observed during the time of the clamp. Although these data imply a potential role of ICAM in modifying EVs for platelet interaction of endothelial cells damage and/or vascular integrity, it remains that ICAM did not directly associate with central hemodynamics. This suggests that adhesion molecules do not play direct role in insulin‐mediated aortic waveform reductions.

Although no work exists in humans with obesity/MetS, in vitro work under hyperglycemic conditions show that platelet, leukocyte, and endothelial EVs impair acetyl‐choline mediated endothelial‐dependent relaxation of carotid arteries and aorta, in part through, reduced eNOS and increased caveolin‐1 (Ishida et al., 2016; Martin et al., 2004). Moreover, endothelial EVs incubated with hyperglycemia increase pro‐coagulant activity ~300% and stimulate HUVEC ROS production by ~250% (Burger et al., 2017). However, others propose that EVs are modified by obesity to promote angiogenesis and improve heart function (Icli et al., 2013), suggesting that compensatory mechanisms in obesity/MetS may exist to minimize vascular dysfunction. Herein, we show for the first time that enriched EVs preps from a healthy control participants augment the effects of insulin on arterial reactivity EVs from participants with MetS blunt this effect. This observation is only preliminary and should be interpreted with caution. Nonetheless, the findings are consistent with the view that EVs likely contain cargo, or are associated with cargo, that are modified with insulin and diseased states that independently and/or together modify vascular function. In fact, this work is consistent with a prior study by our group in which enriched EV preps from normotensive humans retrained vasodilation of mouse arteries during the fasting state that was not observed in people with hypertension (Good et al., 2020). Therefore, additional work is warranted to elucidate the mechanism(s) (e.g. miRNA, NO, etc.) by which these EV enriched preps, or “cleaned EV preps” without co‐sedimented proteins, influence endothelial function and vascular insulin sensitivity to combat disease progression.

Physical activity modifies EV count in relation to cardiometabolic health (Eichner, Erdbrügger, & Malin, 2018). In fact, we recently showed that people with obesity who have very poor physical fitness (VO_2_max = 15.4 ± 0.6 ml/kg/min) had elevated EVs compared to those who were slightly more fit (VO_2_max = 25.9 ± 3.0 ml/kg/min) despite similar age and body composition (Eichner, Gilbertson, et al., 2018). Interestingly, a randomized prospective exercise trial we conducted comparing interval to continuous exercise for 2‐weeks in older adults who had prediabetes showed divergent responses, whereby interval exercise lowered, but continuous exercise increased, EVs in direct relation to gains in VO_2_max (Eichner et al., 2020). This observation is somewhat consistent with prior exercise work suggesting EVs increase in the immediate post‐exercise period, which was suggested to favor angiogenesis (Li et al., 2022; Nederveen et al., 2021; Siqueira et al., 2021; Vechetti et al., 2021). While this prior work has focused on exercise, less data are available testing roles of physical activity and/or sedentary behavior. In this investigation, although we observed no relationship between EVs and sedentary time or time spent in light physical activity, it is possible our subset sample (*n* = 28) was not large enough to capture a significant relationship (data not shown).

There are limitations to the current study that warrant discussion. The study population consisted of mostly white females and thus the generalizability of the study to other ethnic groups and/or sex/genders is dampened. We did not include a healthy control for clamp investigation, and as such, our work is delimited to MetS. However, it is worth noting that these individuals responded to insulin by lowering AIx75 and AP despite having low peripheral insulin sensitivity. This suggests individuals were insulin sensitive at the vasculature level to some extent and enables the assessment of insulin‐stimulated reductions in EV. Nonetheless, we did observe larger reductions in response to insulin among those people with MetS with lower disease severity and our ex vivo data suggest EVs from disease states may uniquely influence vascular function. Thus, additional work is needed in healthy controls. Although AIx75 is used as a surrogate of arterial stiffness, it is important to acknowledge pulse wave velocity measurements were not assessed herein, nor were other measures of endothelial function (e.g. FMD). We also did not include EVs from other cell types (e.g. skeletal muscle or adipose) within our analysis. Instead, we focused on circulating immune (leukocytes/platelets) and vasculature (endothelial) related EVs. Likewise, EVs for the myography assays were collected in the fasted state, thus we are unable to determine if EVs response to insulin could potentially alter the different dilatory response seen between the two groups of EVs collected from healthy and MetS adults, respectively. Future research should consider investigating EVs of other cell types to enhance “cross‐talk” understandings in relation to insulin sensitivity. In addition, enriched EVs were isolated from starting volume in pressure myography assays due to limited availability, and we did not have material to perform EV counting with NTA. However, our volumetric particle counting with flow cytometry allows to assess accurately EV/particle counts. Lastly, our flocytometric detection of EVs does not allow to assess smaller EVs <200 nm, therefore our analysis does not take small EVs into account. This is work for future analysis. Nevertheless, we found correlations which are clinically meaningful with medium and large size EVs supporting that these should not be neglected either.

In conclusion, insulin significantly decreased EVs in participants with MetS along with decreases in aortic waveforms and vascular inflammation. In addition, EVs correlated with indices of peripheral and adipose insulin sensitivity, independent of VAT. These findings strengthen the growing literature that EVs maybe a novel mechanism by which interorgan communication alters physiology to promote insulin resistance and chronic disease progression. However, the specific study was not designed to determine the specific cargo of the EVs, there for it cannot be determined the specific mechanism by which the EVs are affecting the ex vivo vasculature. Additional work is warranted to determine how pure EV preps specifically (e.g. cargo) impact metabolic and vasculature physiology as well as how lifestyle modification, with and without pharmacotherapy, may affect EV‐mediated MetS and related comorbidities.

## AUTHOR CONTRIBUTIONS

Steven K. Malin conceptualized the study. Tristan J. Ragland, Emily M. Heiston, Anna Ballantyne, Nathan R. Stewart, Sabrina La Salvia, Luca Musante, Melissa A. Luse, Brant E. Isakson, Uta Erdbrügger, and Steven K. Malin contributed to data collection. Tristan J. Ragland and Uta Erdbrügger take responsibility for statistical analysis. Tristan J. Ragland and Steven K. Malin co‐wrote the original draft, while all others provided edits. All authors have read and agreed to the final published version of the manuscript.

## FUNDING INFORMATION

Funding for this work was supported by National Institutes of Health RO1‐HL130296 (SKM).

## CONFLICT OF INTEREST

None.

## DISCLOSURES

None to be reported.

## ETHICS STATEMENT

This study was conducted in accordance with The Declaration of Helsinki (1964), except for registration in database.

## CLINICAL TRIALS REGISTRATION

NCT03355469.

## Supporting information


Figure S1

Figure S2

Table S1
