# Supplementary material for: Extracellular vesicles and insulin‐mediated vascular function in metabolic syndrome
Source: Physiol Rep. 2023 Jan 3;11(1):e15530. doi: 10.14814/phy2.15530 (PMC9810789; doi:10.14814/phy2.15530)
Supplement: Supplementary file 1 — Figure S1 [file PHY2-11-e15530-s003.docx]

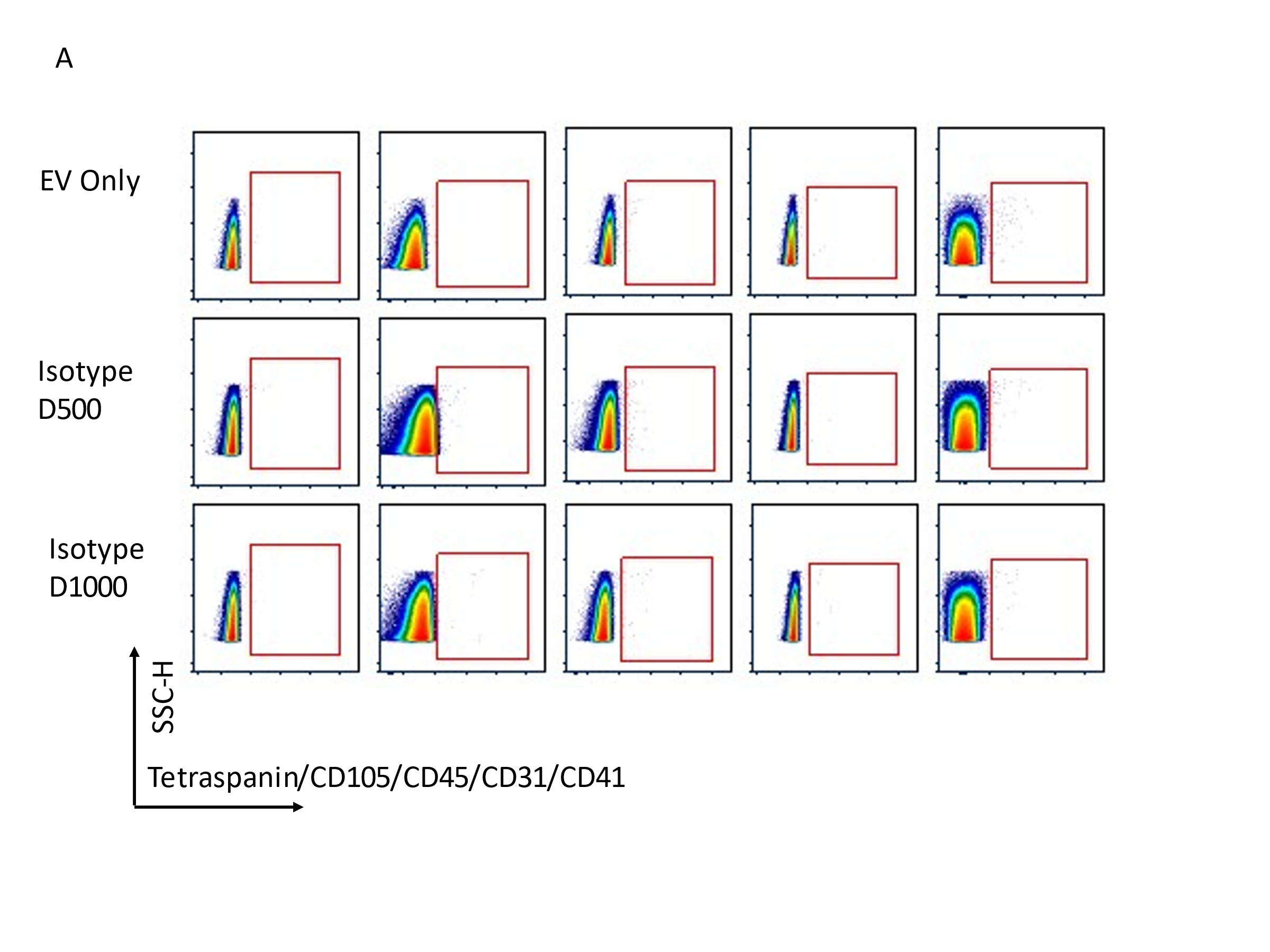
***Figure S1***. Before flow acquisition each sample is diluted with filtered HEPES buffer in different volumes to rule out effects of co-incidence (“swarming”).

Note: In this supplemental figure, we show in Fig. A and B flow blots of examples for an Isotype control mix and EV samples without fluorescent labeling (EV Only), Isotype control mix at dilution 1:500 (D500) and 1:1000 (D1000), EVs with antibody mix (EV P17) in 2 dilutions (EV P17 D500 and EV P17 D1000) and EVs with antibodies also treated with detergent (EV P17 DET D500 and EV P17 DET D500). C summarizes the actual counts for each plot and demonstrates that there is no change in the Median particle count between different dilutions. However, there is a significant reduction in counts with use of detergents.
