# Supplementary material for: Extracellular vesicles and insulin‐mediated vascular function in metabolic syndrome
Source: Physiol Rep. 2023 Jan 3;11(1):e15530. doi: 10.14814/phy2.15530 (PMC9810789; doi:10.14814/phy2.15530)
Supplement: Supplementary file 2 — Figure S2 [file PHY2-11-e15530-s001.docx]

***Figure S2***. Manual gating strategy.


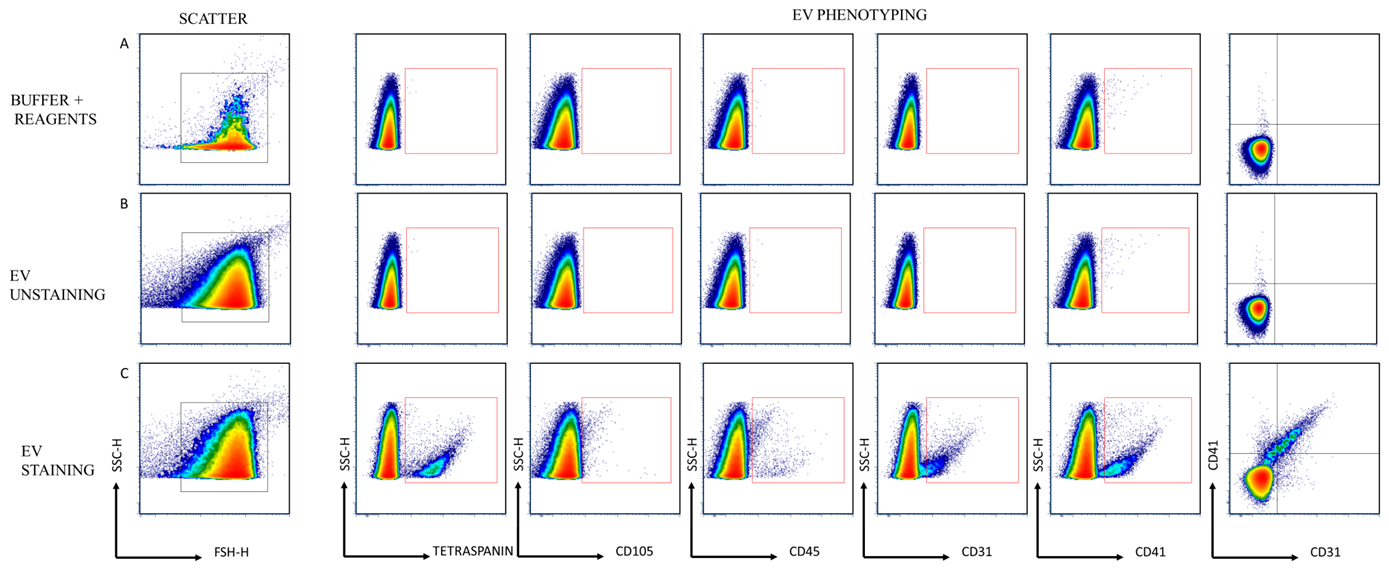


Note: Scatter was based on FSC-H/SSC-H properties. Arrows are used to visualize x and y across plots. Letters A, B, and C are used to call attention to the gating strategy. A) Buffer plus Reagents. B) EV unstaining. C) EV staining. EV phenotyping was performed for the main EV subsets.
