# Supplementary material for: Extracellular vesicles and insulin‐mediated vascular function in metabolic syndrome
Source: Physiol Rep. 2023 Jan 3;11(1):e15530. doi: 10.14814/phy2.15530 (PMC9810789; doi:10.14814/phy2.15530)
Supplement: Supplementary file 3 — Table S1 [file PHY2-11-e15530-s002.docx]

***Table S1***. MIFlowCyt-EV check list.

| **Framework Criteria** | **What to report** | **Please complete each criterion** |  |
| --- | --- | --- | --- |
| 1.1 Preanalytical variables conforming to MISEV guidelines. | Preanalytical variables relating to EV sample including source, collection, isolation, storage, and any others relevant and available in the performed study. | Plasma EV sample were enriched by centrifugation |  |
| 1.2 Experimental design according to MIFlowCyt guidelines. | EV-FC manuscripts should provide a brief description of the experimental aim, keywords, and variables for the performed FC experiment(s) using MIFlowCyt checklist criteria: 1.1, 1.2, and 1.3, respectively. Template found at www.evflowcytometry.org. | Detectable EV recovery was measured using flow cytometry |  |
| 2.1 Sample staining details | State any steps relating to the staining of samples. Along with the method used for staining, provide relevant reagent descriptions as listed in MIFlowCyt guidelines (Section 2.4 Fluorescence Reagent(s) Descriptions). | Reported in the manuscript | 0.25 µL of anti CD9 conjugated with FITC (BD, clone HI9a Cat number 312104), anti CD63 conjugated with FITC (BD, clone H5C6 Cat number 353006), anti CD81 conjugated with FITC (BD, clone 5A6 Cat number 349504)) ); 2 µL anti CD45 conjugated with PE-Dazzle 594 (BD, clone HI30 Cat number 304052); 1 µL of anti CD105 conjugated with PE (BD, clone 43A3 Cat number 323206), 1 µL of anti CD31 conjugated with AF647 (BD, clone WM59 Cat number 303112) and 1 µL of anti CD41 conjugated with Pacific Blue(BD, Clone HIP8 Cat number 303714). All the antibodies were purchased from Biolegend including isotype controls diluted and processed likewise as follow. The antibody cocktails were diluted to 20 µL with HEPES Buffer (10 mM HEPES pH7.4 and 0.15 M NaCl) filtered through a 0.1 μm syringe filter (Minisart PES syringe filter code 16553K, Sartorious) and centrifuge for 1 hour at 21.130g (max speed of the centrifuge: 524/5424 R-Rotor FA-45-24-11, Eppendorf). EV pellets P17 were resolubilized in 30 µL of HEPES buffer and stained with 20 µL of centrifuge antibody mix over night at RT and in dark condition. |
| 2.2 Sample washing details | State any steps relating to the washing of samples. | Not applicable |  |
| 2.3 Sample dilution details | All methods and steps relating to sample dilution. | Reported in the manuscript , see supplemental Fig S1 | sample was diluted with filtered HEPES buffer in different volume (D:500; D1000; D:2000) |
| 3.1 Buffer alone controls. | State whether a buffer-only control was analyzed at the same settings and during the same experiment as the samples of interest. If utilized it is recommended that all samples be recorded for a consistent set period of time e.g. 5 minutes, rather than stopping analysis at a set recorded event count e.g. 100,000 events. This allows comparisons of total particle counts between controls and samples. | HEPES buffer control was included |  |
| 3.2 Buffer with reagent controls. | State whether a buffer with reagent control was analyzed at the same settings, same concentrations, and during the same experiment as the samples of interest. If used state what the results were. | HEPES buffer plus antibodies mix was included. Number of events recorded was negligible and subtracted from the sample reading |  |
| 3.3 Unstained controls. | State whether unstained control samples were analyzed at the same settings and during the same experiment as stained samples. If used, state what the results were, preferably in standard units. | HEPES buffer control was included |  |
| 3.4 Isotype controls. | The use of isotype controls is applicable to immunofluorescence labelling only. State whether isotype controls were analyzed at the same settings and during the same experiment as stained samples. If utilized, state which antibody they are matched to, the concentration used, and what the results were (Section 4.2, 4.3, 4.4). Due to conjugation differences between manufacturers if should be stated if the isotype controls are from the same manufacturer as the matched antibodies. | Isotype controls were included. They were purchased from Biolegend and used at the same working concentration. Mouse IgG1, κ-FITC clone MOPC-21 cat number 981802; Mouse IgG1, κ-Pacific Blue clone MOPC-21 cat number 981812; Mouse IgG1, κ-AF647 clone MOPC-21 cat number 400130; Mouse IgG1, κ-PE Dazzle 594 clone MOPC-21 cat number 981814 and Mouse IgG1, κ-PE clone MOPC-21 cat number 400112 |  |
| 3.5 Single-stained controls. | State whether single-stained controls were included. If used state whether the single-stained controls were recorded using the same settings, dilutions, and during the same experiment as stained samples and state what the results were, preferably in standard units (Section 4.2, 4.3, 4.4). | Single stain controls were included and used as reference controls for the unmixing. |  |
| 3.6 Procedural controls. | State whether procedural controls were included. If used, state the procedure and if the procedural controls were acquired at the same settings and during the same experiment as stained samples. | The same healthy plasma sample control was used and process according to the protocol in each experiment to check inter day variability. |  |
| 3.7 Serial dilutions. | State whether serial dilutions were performed on samples and note the dilution range and manner of testing. The fluorescence and/or scatter signal intensity would ideally be reported in standard units (see Section 4.3, 4.4) but arbitrary units can also be used. This data is best reported by plotting the recorded number events/concentration over a set period of time at different sample dilution. The median fluorescence intensity at each of the dilutions should also ideally be plotted on the same or a separate plot. | Samples were run at a serial dilution to establish the concentration that produced a single EV detection and this happened when abort rate was less than the 10 percent of the count rate. This parameter was used to dilute each sample to avoid swarming. See supplemental figure S1. |  |
| 3.8. Detergent treated EV-samples | State whether samples were detergent treated to assess lability. If utilized, state what detergent was used, the end concentration of the detergent, and what the results were of the lysis. | Ripa buffer was used to lyse the EV. The concentration dropped to less than 30 percent after incubation for 30 minutes at room temperature at the same dilution. See supplemental figure S1. |  |
| 4.1 Trigger Channel(s) and Threshold(s). | The trigger channel(s) and threshold(s) used for event detection. Preferably, the fluorescence calibration (Section 4.3) and/or scatter calibration (Section 4.4) should be used in order to report the trigger channel(s) and threshold(s) in standardized units. | EV analyses were carried out using a 405 SSC trigger and the threshold set at 500 arbitrary unit |  |
| 4.2 Flow Rate / Volumetric quantification. | State if the flow rate was quantified/validated and if so, report the result and how they were obtained. | Samples were run at the lowest flow rate setting. Acquisition volume was measured using the internal flow rate sensor of the cytometer. |  |
| 4.3 Fluorescence Calibration. | State whether fluorescence calibration was implemented, and if so, report the materials and methods used, catalogue numbers, lot numbers, and supplied reference units for the standards. Fluorescence parameters may be reported in standardized units of MESF, ERF, or ABC beads. The type of regression used, and the resulting scatter plot of arbitrary data vs standard data for the reference particles should be supplied. | MESF beads for FITC (Bangs Laboratory Catalogue Number 555A, Lot number 12690), PE (Bangs Laboratory Catalogue Number827A, Lot number 12845) and AF647 (Bangs Laboratory Catalogue Number 647A, Lot number 12929) were used. |  |
| 4.4 Light Scatter Calibration. | State whether and how light scatter calibration was implemented. Light scatter parameters may be reported in standardized units of nm2, along with information required to reproduce the model. | Light scatter calibration was not performed due to no Light scatter parameters being used. |  |
| 5.1 EV diameter/surface area/volume approximation. | State whether and how EV diameter, surface area, and/or volume has been calculated using FC measurements. | Not applicable |  |
| 5.2 EV refractive index approximation. | State whether the EV refractive index has been approximated and how this was done. | Not applicable |  |
| 5.3 EV epitope number approximation. | State whether EV epitope number has been approximated, and if so, how it was approximated. | Not applicable |  |
| 6.1 Completion of MIFlowCyt checklist. | Complete MIFlowCyt checklist criteria 1 to 4 using the MIFlowCyt guidelines. Template found at www.evflowcytometry.org. | Complete |  |
| 6.2 Calibrated channel detection range | If fluorescence or scatter calibration has been carried out, authors should state whether the upper and lower limits of a calibrated detection channel were calculated in standardized units. This can be done by converting the arbitrary unit scale to a calibrated scaled, as discussed in Section 4.3 and 4.4, and providing the highest unit on this scale and the lowest detectable unit above the unstained population. The lowest unit at which a population is deemed ‘positive’ can be determined a variety of ways, including reporting the 99th percentile measurement unit of the unstained population for fluorescence. The chosen method for determining at what unit an event was deemed positive should be clearly outlined. | Done |  |
| 6.3 EV number/concentration. | State whether EV number/concentration has been reported. If calculated, it is preferable to report EV number/concentration in a standardized manner, stating the number/concentration between a set detection range. | Use of the volumetric methods. |  |
| 6.4 EV brightness. | When applicable, state the method by which the brightness of EVs is reported in standardized units of scatter and/or fluorescence. | Not applicable |  |
| 7.1. Sharing of data to a public repository. | Provide a link to the experimental data in a public data repository. | Uploaded in flowrepository.com | Repository ID: FR-FCM-Z4PZ |
